# Convergent occurrence of the developmental hourglass in plant and animal embryogenesis?

**DOI:** 10.1093/aob/mcw024

**Published:** 2016-03-24

**Authors:** Andrew G. Cridge, Peter K. Dearden, Lynette R. Brownfield

**Affiliations:** ^1^Laboratory for Evolution and Development, Genetics Otago and Department of Biochemistry, University of Otago, Dunedin, 9054, New Zealand and; ^2^Department of Biochemistry, University of Otago, Dunedin, 9054, New Zealand

**Keywords:** Embryogenesis, developmental hourglass, convergent evolution, developmental networks, comparative transcriptomic analysis, gene expression

## Abstract

**Background** The remarkable similarity of animal embryos at particular stages of development led to the proposal of a developmental hourglass. In this model, early events in development are less conserved across species but lead to a highly conserved ‘phylotypic period’. Beyond this stage, the model suggests that development once again becomes less conserved, leading to the diversity of forms. Recent comparative studies of gene expression in animal groups have provided strong support for the hourglass model. How and why might such an hourglass pattern be generated? More importantly, how might early acting events in development evolve while still maintaining a later conserved stage?

**Scope** The discovery that an hourglass pattern may also exist in the embryogenesis of plants provides comparative data that may help us explain this phenomenon. Whether the developmental hourglass occurs in plants, and what this means for our understanding of embryogenesis in plants and animals is discussed. Models by which conserved early-acting genes might change their functional role in the evolution of gene networks, how networks buffer these changes, and how that might constrain, or confer diversity, of the body plan are also discused.

**Conclusions** Evidence of a morphological and molecular hourglass in plant and animal embryogenesis suggests convergent evolution. This convergence is likely due to developmental constraints imposed upon embryogenesis by the need to produce a viable embryo with an established body plan, controlled by the architecture of the underlying gene regulatory networks. As the body plan is largely laid down during the middle phases of embryo development in plants and animals, then it is perhaps not surprising this stage represents the narrow waist of the hourglass where the gene regulatory networks are the oldest and most robust and integrated, limiting species diversity and constraining morphological space.

## EMBRYOGENESIS

Embryogenesis describes the development of a single fertilized cell into a mature embryo, in which the basic tissue types and body plan for that organism are established. It begins with the fusion of male and female gametes to create the single-celled zygote, with the potential to form a whole organism through cell division and expansion, along with cell type-specific specification, differentiation and maturation. While embryogenesis does not occur in single-celled eukaryotes, it has evolved independently in two of the major multicellular lineages, animals (Animalia) and land plants (Embryophyta) (Meyerowitz, 2002). Producing multicellular offspring through embryogenesis enables the basic body plan to be established, while nutrition and protection provided by the mother increase the chance of survival. In seed plants, embryogenesis occurs as part of seed development and so aids in the dispersal of offspring.

While evolutionary change that affects the embryonic process does occur, ultimately viable offspring must be produced, presumably constraining the total variation that may arise. In the case of the developmental hourglass model, where early events in development are less conserved across species, but lead to a highly conserved ‘phylotypic period’ during mid-embryogenesis before again diverging, how do early-acting events in development evolve whilst providing the scaffold on which all of embryogenesis is built? Here we compare morphological and molecular processes that occur during plant and animal embryogenesis, suggesting that these independently evolved processes both show an hourglass pattern, to draw out general processes that may lead to hourglass models.

## THE HOURGLASS MODEL OF EMBRYOGENESIS IN ANIMALIA

**Fig. 1.**
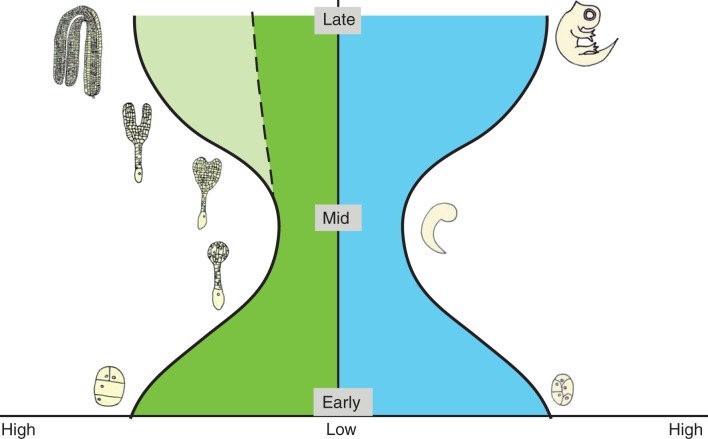
Comparison of the morphological and transcriptome hourglass model between flowering plants and animals. In both panels, embryogenesis proceeds from the bottom to the top (early, mid and late stages), and the width represents the morphological and transcriptome diversity. Left: the hourglass model in flowering plants predicts that the mid-embryonic stage (globular to torpedo) is the most conserved stage. Plant embryogenesis begins with the formation of the zygote, followed by several rounds of mitotic divisions to produce a cluster of cells, the globular embryo. The embryo passes from the globular stage through a transition stage to the heart stage, and finally reaches the torpedo stage, in which all the tissue types and organs have been established. Late-stage embryos lack morphological conservation (dark green area) but exhibit transcriptional variation (light green area). Right: the hourglass model in animals predicts that the morphological ‘phylotypic period’ around the mid-embryonic stages is the most conserved. Animal embryogenesis begins with the formation of the zygote followed by several rounds of mitotic divisions to produce a cluster of cells in which the main axes of the adult morphology are established, and the broad domains of the body plan defined. The subsequent middle period of embryogenesis is also known as the ‘phylotypic period’ as species in common phyla share morphological similarities despite differences in the early stages. In the last stage of embryo development, the limbs, organs, eyes and other structures form, resulting in the final structures of the adult or larvae. By the end of development, the differing growth and patterning trajectories of different species lead to increased divergence and diversity in adult body plans.

Nineteenth century German embryologist Karl Ernst von Baer, from his morphological observations of embryogenesis in a range of animal species, noted that embryos from species in the same phylum often show considerable variation early in embryogenesis, then converge to a similar form during mid-embryogenesis, before diverging again late in embryogenesis (von Baer, 1828). These observations have led to the development of the hourglass model of embryo development in animals (Fig. 1). This model divides metazoan animal embryogenesis into three stages. The first stage encompasses early embryogenesis and begins with the formation of the zygote. During this stage, multiple rounds of mitotic division generate undifferentiated cells, which are then distributed into layers, through gastrulation. Additionally, the main axes of the adult morphology are established, and the broad domains of the body plan defined at this stage. Morphological observations have consistently indicated that these early events can be very different between metazoan species from the same phylum, as shown in insects (Sander, 1975), nematodes (Goldstein *et al.*, 1998) and vertebrates (Irmler *et al.*, 2004). The subsequent middle period of embryogenesis is also known as the ‘phylotypic period’ (Richardson, 1995), defined as the stage at which all body parts are represented in their final positions as undifferentiated cell condensations, or the stage at which organisms within a common phylum show the maximum degree of morphological similarity despite differences in the early stages (Slack *et al.*, 1993). During this stage, patterning along the axes, especially that reflected in *Hox* gene expression (Harding *et al.*, 1985; Akam, 1987), is established. In the last stage of embryo development, the limbs, organs, eyes and other structures form, resulting in the final structures of the adult or larvae. By the end of development, the differing growth and patterning trajectories of different species lead to increased divergence and diversity in adult body plans.

Despite morphological analyses in animals suggesting the existence of the developmental hourglass model, it remained controversial due to the complexity of evaluating evolutionary distances between embryos of diverse species simply by comparison of morphogenesis (Hall, 1997; Richardson *et al.*, 1997; Galis and Metz, 2001; Bininda-Emonds *et al.*, 2003; Irie and Sehara-Fujisawa, 2007; Roux and Robinson-Rechavi, 2008; Comte *et al.*, 2010). Development of comparative transcriptomic analysis has enabled comparison of gene expression at different developmental stages (Domazet-Loso and Tautz, 2010; Kalinka *et al.*, 2010; Irie and Kuratani, 2011; Yanai *et al.*, 2011), thereby bringing a more quantitative methodology to the traditionally qualitative discipline of comparative embryology. By comparing gene expression profiles across species of invertebrate (*Drosophila melanogaster*, *Anopheles gambiae* and *Caenorhabditis elegans*) (Kalinka *et al.*, 2010; Schep and Adryan, 2013), chordate (*Ciona intestinalis*) and vertebrate (*Danio rerio* and *Xenopus tropicalis*) (Schep and Adryan, 2013), it has been possible to address conservation in the different stages of embryo development across a range of measures, including the evolutionary age and sequence divergence of gene expression (Domazet-Loso and Tautz, 2010; Yanai *et al.*, 2011). All of these approaches have provided molecular support for the developmental hourglass model, by highlighting that the highest level of conservation occurs during the ‘middle’ stages of embryogenesis, corresponding roughly to the morphologically conserved phylotypic period.

## THE HOURGLASS MODEL OF EMBRYOGENESIS IN ANGIOSPERMAE

Embryogenesis evolved early and independently in the land plant linage, with the bryophytes producing multicellular embryos. As in animals, the basic body plan in plants is established during embryo development, with the mature embryo containing the tissues and organs required for an adult (West and Harada, 1993; Jurgens *et al.*, 1995; Mordhorst *et al.*, 1997; Chandler *et al.*, 2008; Harada *et al.*, 2010; Peris *et al.*, 2010; Wendrich and Weijers, 2013; ten Hove *et al.*, 2015). This body plan can be considered as a series of elements arranged along two axes: a radial axis and a shoot–root axis. In flowering plants, the radial axis consists of concentric tissue layers containing the three basic tissue types that will form all plant structures. These are, from the outside to the inside, the epidermis, the ground tissue and the central vascular tissue. Along the shoot–root axis is the shoot apical meristem and the cotyledons at the one end, the hypocotyl in the middle and an embryonic root, including a root meristem, at the other end. In seed plants, embryogenesis occurs alongside the processes required for seed development, with the mature embryo neatly packaged in the seed for dispersal.

While the basic body plan is established during plant embryogenesis, the mature embryos of plants are anatomically much less complex than those in animals, and much of the morphological variations observed between plant taxa is established post-embryonically. Due to this reduced complexity, the possible existence of an hourglass pattern of development was largely ignored in plant morphological studies as plant embryogenesis was considered not to generate sufficient morphological diversity (Quint *et al.*, 2012; Drost *et al.*, 2015). However, more recent molecular data imply that flowering plant embryogenesis may also follow an hourglass pattern. Gene expression data from *Arabidopsis thaliana* embryos at a number of stages of development were used to calculate the age and the sequence divergence of the transcriptomic throughout embryonic development (Quint *et al.*, 2012; Drost *et al.*, 2015). Both of these phylotranscriptomic analyses point to an hourglass model, with the mid-embryonic stages (globular to torpedo) (Fig. 1) being the most conserved in terms of gene expression and thus forming the waist of the hourglass, with more divergent early and late phases. With molecular data suggesting that an hourglass pattern of development may be present in plants, it is relevant to review whether there is evidence for a morphological hourglass in plant embryogenesis, albeit one that may be reduced in morphological complexity compared with animals.

There is a long history of morphological studies in plant embryology reviewed in a number of substantial works such as Wardlaw (1955), Gifford and Foster (1989), Johri *et al.* (1992) and Raghavan and Sharma (1995). Our aim here is not to review these works, but rather to highlight certain aspects to ask if there are some similarities on the morphological level between the hourglass models observed in animals and events in plant embryogenesis. The study of plant embryogenesis often encompasses a wide range of events in plant reproduction such as gametogenesis, pollination, fertilization and seed development. Here our focus will be on zygotic embryogenesis and events that occur from the zygote to the mature embryo. As phylotranscriptomic support for an hourglass model is currently only available for the flowering plant *A. thaliana*, the discussion will also be limited to the flowering plants (angiosperms).

### Is there a morphological hourglass in flowering plant embryogenesis?

Embryo development in flowering plants can be divided into three stages (Raghavan and Sharma, 1995; Harada *et al.*, 2010; Peris *et al.*, 2010; ten Hove *et al.*, 2015). The first stage involves the development of the zygote followed by multiple rounds of mitotic divisions without a large increase in total size, to produce a ball of cells known as the globular embryo. While the cells in the globular embryo are largely undifferentiated, both the apical–basal axis and the radial axis are established by this stage. Morphological surveys have revealed that there is considerable diversity in the pattern of cell divisions, or segmentation, to generate the globular embryo (Wardlaw, 1955; Johri *et al.*, 1992; Raghavan and Sharma, 1995). Based on the sequence and orientation of cell division, six distinct cleavage patterns have been described (Johansen, 1950; Maheshwarl, 1950) (Table 1). Interestingly, these different patterns are distributed across flowering plant taxa and show little relationship to angiosperm taxonomy (Wardlaw, 1955). Similar patterns are found in distantly related families; for example, the Onagrad and Asterad types are found in the orders from the basal eudicots, the core eudicots (both Rosids and Asterad groups) and the monocots (Table 1). Additionally, different patterns are found in related species, such as the order Ranunculales containing genera that display the Onagrad, Solanad and Caryophyllad patterns of early embryo development (Table 1). Some species also display more than one pattern (Caryophyllales), and some species, such as cotton (*Gossypium hirsutum*) and maize (*Zea mays*), do not have a precise pattern of mitotic division, with the early division patterns appearing to be random (Pollock and Jensen, 1964; Poethig, 1987). The morphological surveys of early plant embryogenesis thus show considerable diversity in the segmentation pattern as plant embryos progress to the globular stage, and this diversity is not tightly associated with phylogeny. This morphological diversity is reminiscent of the diversity observed in early embryos in animals, providing the wide base to the hourglass (Fig. 1).

**Table 1.** The different patterns of early embryo segregation in plants and some of the Orders in which the patterns have been reported

Type*	Order reported^†^	Major clade
Onagrad (Crucifer)	Myrtales	Core eudicot – Rosids
	Lamiales	Core eudicot – Asterids
	Brassicales	Core eudicot – Rosids
	Malpighiales	Core eudicot – Rosids
	Fabales	Core eudicot – Rosids
	Ranunculales	Basal eudicot
	Sapindales	Core eudicot – Rosids
	Asparagales	Monocot
	Liliales	Monocot
	Poales	Monocot
Asterad	Asterales	Core eudicot – Asterids
	Geraniales	Core eudicot – Rosids
	Lamiales	Core eudicot – Asterids
	Oxilidales	Core eudicot – Rosids
	Caryophyllales	Basal eudicot
	Rosales	Core eudicot – Rosids
	Liliales	Monocot
	Poales	Monocot
Solanad	Solanales	Core eudicot – Asterids
	Apiales	Core eudicot – Asterids
	Piperales	Basal angiosperm
	Malpighiales	Core eudicot – Rosids
	Ranunculales	Basal eudicot
	Gentianales	Core eudicot – Asterids
Chenopodiad	Caryophyllales	Basal eudicot
	Ericales	Core eudicot – Asterids
	Boraginaceae (unplaced order Lamiales?)	Core eudicot – Asterids
Caryophyllad	Caryophyllales	Basal eudicot
	Saxifragales	Basal eudicot
	Ranunculales	Basal eudicot
	Fabales	Core eudicot – Rosids
	Ericales	Core eudicot – Asterids
	Alismatales	Monocot
Piperad	Piperales	Basal angiosperm
	Santalales	Basal eudicot
	Asterales	Core eudicot – Asterids
	Malpighiales	Core eudicot – Rosids
	Dipsacales	Core eudicot – Asterids

* As described in Johansen (1950).

^†^ Based on information in Wardlaw (1955) and Raghaven and Sharma (1995).

As in animals, it is during the middle phase of embryogenesis that patterning occurs in plants, with the basic body plan being laid down (West and Harada, 1993; Mordhorst *et al.*, 1997; Chandler *et al.*, 2008; Harada *et al.*, 2010; Peris *et al.*, 2010; Wendrich and Weijers, 2013). Despite the diversity in cell division patterns in the early stages, the basic architecture established in the middle phase is similar in the major plant taxa (Wardlaw, 1955; Peris *et al.*, 2010). In eudicots, the middle phase covers the globular stage to the torpedo stage. It begins with the transition from the globular embryo to the heart stage as cotelydons begin to form at two lateral zones at the apical domain, altering the symmetry from radial to bilateral. During this middle phase, the root and shoot meristems are specified, the hypocotyl becomes apparent and tissue specification takes place, with the precursors of the vascular tissue forming. There is also a considerable increase in size. Notably, the morphology of the embryo during this stage is similar throughout the eudicots regardless of the early segmentation pattern (Wardlaw, 1955; Mordhorst *et al.*, 1997). For example, Mordhorst *et al.* (1997) noted the high level of similarity in the middle stages between *A. thaliana* (Brassicales), a Rosid that displays the Onagrad pattern in early embryogenesis, and carrot (*Daucus carota*, Apiales), an Asterid that displays a Solanad pattern in early embryogenesis. The morphology of cotton embryos is also similar to that of other dicots in the middle phase, despite the random nature of the early pattern of cell division (Pollock and Jensen, 1964).

The morphology of monocot embryos, however, becomes dramatically different from those of eudicots during the middle phase (Raghavan and Sharma, 1995; Mordhorst *et al.*, 1997; Chandler *et al.*, 2008). The major differences relate to the formation of a single cotyledon, the shoot meristem forming laterally rather than apically, and the resultant shoot–root axis not aligning with the apical–basal axis established early in development. There is also greater variation amongst monocot embryos than the dicots, with the grasses being markedly different, with additional tissues such as the absorptive scutellum and the protective coleoptile and coleorhiza covering the young shoot and root (Raghavan and Sharma, 1995). Thus comparative morphological studies indicate that there is reduced morphological variation within plant taxa in the middle phase of plant embryogenesis compared with the early phase. This reduction in morphological diversity is similar to the narrowing of the hourglass to the constricted waist seen in morphological analyses of animal embryogenesis (Fig. 1). While this similarity to the hourglass model has not been widely commented on previously, the observation that there is a reduction in morphological diversity during plant embryogenesis has been noted (Wardlaw, 1955; Yadegari and Goldberg, 1997; Peris *et al.*, 2010).

In animals, the waist of the hourglass relates to the ‘phylotypic’ stage where related taxa are more similar than at earlier and later stages based on both morphological and molecular data. The middle period of plant embryogenesis was also defined as the phylotypic stage based on phytotransciptomic data (Quint *et al.*, 2012; Drost *et al.*, 2015). This then raises the question of whether the middle period of plant embryogenesis can be considered ‘phylotypic’ based on morphology. On a broad basis, the answer appears to be yes, as the differences in morphology at this stage are divided along phylogenetic lines, with the major differences being between the monophyletic eudicots and the monocots. Furthermore additional variations, such as the scutellum, coleoptile and coleorhiza in the grasses, are only found confined to monophyletic groups. Thus, in the middle phase, embryos from related taxa appear similar, with variation between taxa, meaning that this phase could be described as ‘phylotypic’ in flowering plants based on morphology as well as molecular studies.

The events occurring in the last phase of embryogenesis are quite distinct in plants and animals. In animals, the body plan is built upon as the major organs form, and embryos from related taxa again diversify in morphology. In flowering plants, the third phase in embryogenesis is a maturation phase and does not involve the further elaboration of the body plan (West and Harada, 1993; Harada *et al.*, 2010). The main noticeable change in the embryo is an expansion, with little change in the overall architecture. Other events at this stage relate to the packaging of the embryo into a seed for dispersal such as preparation for desiccation, metabolic quiescence and nutrient storage. Thus, in the third stage of embryo development in flowering plants, the morphological hourglass pattern is not followed. Rather than the divergence of morphology and the widening of the hourglass observed in animal embryo morphology, the range of plant diversity remains similar, forming a funnel-like shape rather than an hourglass (Fig. 1). The similarity in embryo morphology remains high within related taxa, so this stage could also be considered ‘phylotypic’.

Overall it seems that comparative morphology of embryogenesis of flowering plants does show some similarity to the hourglass model described for animal embryogenesis (Fig. 1). There is a high degree of variation in the early stages, and this variation is found within related taxa. The level of variation is then reduced in the middle stages, most notably within related taxa. Thus, these stages reconstitute the hourglass pattern, beginning broad and then narrowing to the waist. However, the later divergence seen in animal embryos does not occur in the flowering plants. While there is some similarity to the hourglass model, it would be speculative to say that plant embryogenesis follows the hourglass model based purely on morphology. However, if we couple the morphological data with the phylotranscriptomic data (Quint *et al.*, 2012; Drost *et al.*, 2015), there is much stronger evidence that plant embryogenesis follows the hourglass model. This observation is intriguing as embryogenesis evolved independently in plants and animals, suggesting that the hourglass pattern has evolved twice. This raises the question – is this just coincidence or a case of convergent evolution? If it were a case of convergent evolution, this would point to the existence of evolutionary factors that favour the hourglass pattern of embryo development. To try and address if this is convergent evolution, we must consider the interplay between evolutionary and developmental processes, and ask if there are limits or constraints on taxonomic diversity or morphological disparity during embryogenesis. We discuss whether the conserved waist of the hourglass model may be required for spatio-temporal organization and differentiation of complex multicellular life (Quint *et al.*, 2012).

## CONVERGENT EVOLUTION OF THE HOURGLASS PATTERN OF EMBRYO DEVELOPMENT

Developmental constraints, defined as ‘…the non-production of variant phenotypes caused by the nature of the developmental system’ (Arthur and Farrow, 1999), imply that the architecture of any given developmental system precludes or biases some developmental outcomes. Although the relationship between the mechanisms that allow variation and those that constrain outcomes is unclear, what is apparent is that these constraints are universal, in that they limit the evolution that can occur, as essentially there are only some changes to the developmental pathways that ‘work’ and a multitude of ‘variations’ that ‘don’t work’ and are non-viable, resulting in constraints on radial evolution. These events in embryogenesis are constrained by the absolute requirement for the generation of a mature embryo, with all the tissues and organs required to mature into a reproductively fit adult. Any alterations in embryo development that impact negatively upon post-embryonic events would be selected against. Convergent evolution of an hourglass pattern of embryo development in animals and plants would imply that the mid-embryonic stages are particularly constrained, and resistant to evolutionary change, compared with the more flexible early and late stages. By considering what general events occur at each stage of embryo development at the morphological and molecular levels in plants and animals, we explore why the hourglass model may fit in these independently evolved ontogenies.

### Constraints in early embryo development

**Fig. 2.**
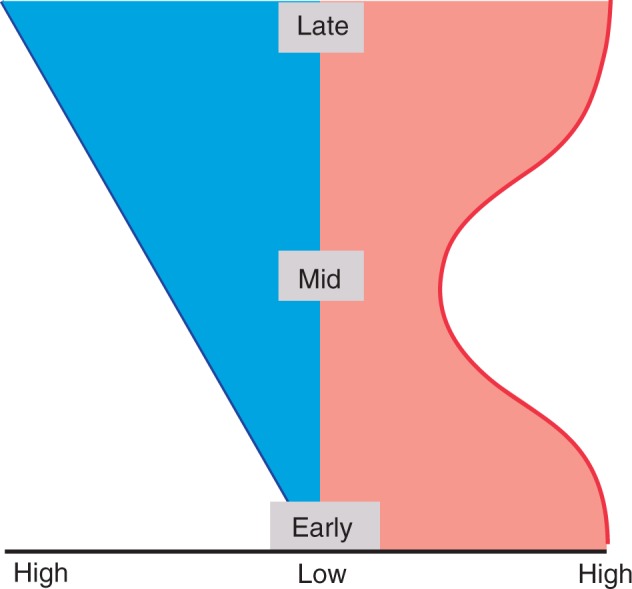
The two major hypotheses about how developmental processes are conserved. In both models, embryogenesis proceeds from the bottom to the top (early, mid and late stages), and the width represents the phylogenetic diversity of developmental processes, which are deduced from morphological similarities. Left: the funnel-like model predicts conservation at the earliest embryonic stage. During embryogenesis, diversity increases additively and progressively, leading to the diversity of forms. Right: the hourglass model predicts conservation of the ‘phylotypic period’. During embryogenesis, early events in development are less conserved across species but lead to a highly conserved ‘phylotypic period’. Beyond this stage, the model suggests that development once again becomes less conserved, leading to the diversity of forms.

We might presume that the earliest stages of embryo development would be the most constrained, as it could be expected that alterations early in development would be more likely to have widespread downstream effects. As development progresses, alterations would then be more widely tolerated, in turn promoting diversity. This funnel-like model predicts that the highest conservation would occur at the earliest stages of development (Fig. 2) (Raff, 1996). Conservation of early embryogenesis is, however, not seen on the morphological or molecular levels in plants and animals, implying that this early stage is less constrained that the subsequent mid-embryonic stages. This observation is intriguing as it suggests a measure of plasticity previously not considered permissible, considering the importance of establishing the correct body plan in the mature embryo

To understand why this stage might be less constrained, we need to consider the events that occur during early embryogenesis. The beginning of embryogenesis in both plants and animals involves the mitotic division of the zygote to produce a multicellular embryo. During this stage, maternal factors and early-expressed zygotic genes establish the major axes of the embryo, while the individual cells are largely undifferentiated and maintain the potential to form multiple cell types. The expression of the genes involved in this stage of embryogenesis is less conserved than in the following stages in vertebrates, invertebrates and flowering plants (Davis *et al.*, 2005; Hazkani-Covo *et al.*, 2005; Demuth and Wade, 2007; Irie and Sehara-Fujisawa, 2007; Cruickshank and Wade, 2008; Artieri *et al.*, 2009; Domazet-Loso and Tautz, 2010; Kalinka *et al.*, 2010; Irie and Kuratani, 2011; Yanai *et al.*, 2011; Quint *et al.*, 2012). This suggests that the earliest periods of development are divergent in terms of not only morphology but also gene expression and protein sequence evolution. The implication is that as long as the gene expression pathways can establish the major axes of the embryo, then development can proceed. The expression, function or sequence of the early-acting genes can change, as long as the outcomes of the genetic pathways they are involved in do not. This allows the evolution of variation in early embryogenesis on which selection can ultimately be applied. Given that axes can be determined through various and subtle asymmetries in a single cell (e.g. reviewed for insects in Peel *et al.*, 2005), it is possible that developmental drift, between a range of potential axis-forming signals, is possible.

Not only is the expression of genes important, the processes and networks that regulate this expression also require consideration. A combination of interactions forms a hierarchical developmental network within which several sub-networks control the development of particular body parts and regions (Peter and Davidson, 2011). Yet it is ultimately the properties of the sub-networks that are crucial for determining the nature of the selective constraints acting on particular genes at particular times in development. It is important to note that during early embryogenesis, a relatively small number of genes comprise the developmental networks that determine broad regions of the embryo. For example in *A. thaliana*, the outer cell layer (the protoderm) is marked by the transcription factors ATML1 (*Arabidopsis thaliana* meristem layer 1) and PDF2 (protodermal factor 2) by the globular stage, but these cells are still not differentiated into epidermal cells (ten Hove *et al.*, 2015). It is in the subsequent developmental stage that proteins contribute to the establishment of tight networks leading to epidermal cell differentiation. The same can apply to animals; for example, during *Drosophila* segmentation, early stages are marked out by the broad domains of the *gap* genes, combinations of which eventually lead to more refined patterns and cell differentiation (reviewed in Peel *et al.*, 2005).

Changes in expression, temporal co-ordination or addition of novel components to early gene regulatory networks are tolerated, and this implies that there is significant plasticity in the networks active in early development. As long as a multicellular embryo consisting of largely undifferentiated cells with established axes and broad regulatory domains is created, it does not matter how the embryo reaches this stage. The constraint is the end-product, but that product does not have to be tightly defined. This is consistent with species such as cotton, where early development is somewhat random, yet produces a functional globular embryo (Pollock and Jensen, 1964). This is also supported by observations in *Capsella* that has been widely studied as a model for plant embryogenesis. While its early divisions follow a precise pattern, early morphological deviations from this pattern have been observed but do not appear to impact negatively upon the subsequent developmental stages (Pollock and Jensen, 1964).

### Constraints in mid-embryo development

During the middle stages of embryogenesis, the body plan develops further upon the established axes. In most metazoans, the middle stages of embryogenesis are characterized by the expression of the *Hox* complex of transcription factors, which act to define and regionalize the different areas of the embryo trunk along the anterior/posterior axis of the embryo (Garcia-Fernandez, 2005). This pattern of gene expression is activated by previously acting genes and broad domains (Irish *et al.*, 1989), defines the identity of functional units along the anterior–posterior axis from which the rest of the embryonic structures are built, and forms the basis of different morphologies in each of those units (reviewed in McGinnis and Krumlauf, 1992). It seems that this stage is tightly constrained, perhaps because of the importance of this regionalization in anterior–posterior elongated embryos (Duboule, 1994). A similar situation occurs in plants, with the middle embryonic stages characterized by a number of transcription factors that define regions along the shoot–root axis and cotyledon borders (Chandler *et al.*, 2008; Peris *et al.*, 2010). These transcription factors include the WUSCHEL HOMEOBOX LIKE genes: WUS that is critical for shoot meristem activity, WOX5 involved in root specification, SCARECROW (SCR) expressed in the endodermal layer surrounding the vasculature and CUP-SHAPED COTYLEDON (CUC) which is important for the boundary between the cotyledons and the shoot meristem (Chandler *et al.*, 2008; Peris *et al.*, 2010). It is of note that the temporal and spatial expression of these transcription factors is broadly similar in the eudicot *A. thaliana* and the monocot *Z. mays*, despite the difference in eudicot and monocot embryos at this stage (Chandler *et al.*, 2008). This conservation in transcription factor patterning in both animals and plants highlights the importance of cells differentiating in the correct spatial and temporal pattern during the middle stages of embryogenesis. Such expression patterns in turn require complex signalling and regulatory pathways and networks. How does the need for the production of tissues/differentiated cells, along with the complex pathways that regulate them, influence the evolution of embryo development during this middle period?

It is possible that variation in embryogenesis comes from the evolution of developmental pathways that allow the outputs of the pathways to remain stable while developing new parallel processes that can permit stepwise change to embryogenesis to occur. Although limited in scope and complexity, multiple studies have shown that in developmental evolution, different species use conserved genes in alternative ways (Carroll, 2008; Shubin *et al.*, 2009; Wilson and Dearden, 2009; Wilson *et al.*, 2010), and this altered usage gives rise to morphological variation (Sucena *et al.*, 2003; Prud’homme *et al.*, 2006; Chan *et al.*, 2010). This is especially true for transcription factors that are involved in spatial and temporal regulation of (often) large numbers of other genes (Liang and Biggin, 1998; Li *et al.*, 2008). Both the expression and activity of transcription factors during embryogenesis are tightly regulated. The construction of multicellular organisms relies on complex networks of genes that are regulated by transcription factors that, in turn, regulate other genes. Therefore, networks must themselves change to produce morphological change during evolution (Davidson and Levine, 2008). The complexity of these networks, however, may begin to constrain morphological variation as a new role for a conserved transcription factor may have pleiotropic effects on the integrated biology of the organism. We know little about how such gene networks act, and less about how they evolve in embryogenesis (Goltsev *et al.*, 2007; Maduro, 2009), yet changes to such networks may constrain (Cameron *et al.*, 2005), or confer diversity in, an organism. Genetic networks are robust, due to multiple feedback, feed-forward and cross-regulatory mechanisms (von Dassow *et al.*, 2000; Eldar *et al.*, 2002; Davidson, 2010; Hilgers *et al.*, 2010; Holme, 2011; Gavin-Smyth *et al.*, 2013; Zheng *et al.*, 2013). This makes it probable that changes in expression, mediated by transcription factors, of multiple genes are needed to ‘rewire’ these networks, and thereby change their outcome at the morphological level.

Investigation of expression of orthologous transcription factors at different time points during embryonic development across vertebrate (*D. rerio* and *X. tropicalis*), chordate (*C. intestinalis*) and invertebrate (*D. melanogaster*, *A. gambiae* and *C. elegans*) (Schep and Adryan, 2013) phyla showed very similar transcription factor expression patterns between species. Transcription factor expression increases during the initial stages of development, with C2H2 zinc finger transcription factors over-represented and Homeobox transcription factors under-represented in the early stages, but increasing later in development in all species investigated (Schep and Adryan, 2013).

Mid-embryogenesis is thus characterized by precise co-ordination between growth and patterning, and as such is highly sensitive to perturbations in the sequence of temporal and spatial activation of genes (Duboule, 1994). Taking a more global view of conservation, Raff argued that the complexity of interactions between genes, cells and developmental processes reaches a maximum during mid-embryogenesis when the body plan of the organism is being established (Raff, 1996). Common to both models is the idea that changes during mid-embryogenesis are deleterious in nature due to the properties of the developmental system that are unique to this period. However, to what extent variation at mid-embryogenesis is limited purely by selective constraints, or by the interplay between selective and developmental constraints, is not specified in either model. An example of this is the gene-regulatory network controlling root stele development, in which morphological phenotypes were found to be associated with mutations in only 16 % of the transcription factors tested, whereas molecular or expression phenotypes were identified for 65 % (Brady *et al.*, 2011). Accordingly, the transcriptional network can be affected in a transcription factor mutant despite the absence of a mutant phenotype (van der Graaff *et al.*, 2002; Brady *et al.*, 2011). Therefore, mutations in compensatory genes or changes in the expression of several genes may be needed to allow the creation of a new steady state of the network, resulting in a robust change in morphology.

How do we reconcile changes in the network with the conservation we observe in the developmental hourglass model? The most likely explanation is that the hourglass model represents an evolutionarily constrained central node in the ‘hierarchical’ system structure of the developmental network essential for maintaining core gene networks that regulate essential developmental outcomes, such as an antero-posterior identity of the embryo. Mutations that affect general ‘upstream’ regulators (usually highly connected nodes in a network) of developmental or cellular gene expression are more likely to have pleiotropic effects; therefore, these mutations tend to reduce fitness (Stern and Orgogozo, 2009). In contrast, genes that execute cellular responses downstream often act with other genes in a concerted fashion in basic cellular functions. The expression of these genes needs to be modulated in a co-ordinated manner, usually by transcription factors (Stern and Orgogozo, 2008). Comparative epigenomics in *Oryzias latipes* and *D. rerio* identified conserved *cis*-regulatory nodes active during the phylotypic period in these species. A large proportion of these *cis*-regulatory nodes provided regulatory input to genes encoding transcription factors, suggesting that these regulatory regions represent constrained nodes from essential gene regulator networks operating at the phyotypic period (Tena *et al.*, 2014). Therefore, the time at which a gene is expressed relative to the central node will determine how far a gene can evolve, relative to the genes that control the central node (Stern and Orgogozo, 2009).

Analysis of *D. rerio* and *D. melanogaster* identified that evolutionarily younger transcription factors seem to be more important in later stages of development, whereas evolutionarily older genes are prevalent in the earlier stages of development. Indeed, transcriptional expression of conserved transcription factors, by itself, marked the phylotypic stage of metazoans (de Mendoza *et al.*, 2013). By changing the expression of input–output genes of the node, morphologies can be modulated in a specific context. In line with this, some types of transcription factors were repeatedly (although not exclusively) recruited to modify organ morphologies in a certain manner in plants. One example is the heterotopic expression of KNOX transcription factors resulting in dissected leaf development (Bharathan *et al.*, 2002; Hay and Tsiantis, 2006; Hay and Tsiantis, 2010). Another example is the recurrent recruitment of CYC-type TCP transcription factors in generating monosymmetric flowers (zygomorphic) across distant eudicot lineages (Busch and Zachgo, 2009). This recurrent recruitment might be linked to an ancestral dorsal expression domain in floral meristems that were selectively expanded and/or switched to later stages of organ development in monosymmetric taxa (Preston and Hileman, 2009; Busch *et al.*, 2012).

### Constraints in late embryo development

Late embryogenesis differs markedly between plants and animals. Animal embryos at this stage build on their regionalized axes to produce limbs and organs, elaborating on body plans to produce diverse morphologies. Differences in late embryogenesis produce taxa-specific patterns and morphological variation in adult forms. Such morphological variation is underpinned by selection for the structures of the final form, showing that evolution of this late stage of development can shape and re-shape form to fit the final animal’s environment. At these stages, evolutionary changes in gene expression are presumably less pleiotropic, providing morphological detail rather than fundamental structures on which morphology is built.

In flowering plants, the body plan does not change dramatically during the later stages of embryogenesis, as the basic body plan is elaborated upon post-embryonically. Rather, the embryo expands, and processes such as nutrient accumulation and preparation for dehydration occur as part of seed development (Harada *et al.*, 2010). While flowering plant embryos do not show the morphological variations in the later embryo stages that are seen in animals, a molecular hourglass pattern is observed in *A. thaliana* embryogenesis (Quint *et al.*, 2012; Drost *et al.*, 2015). The transcriptome at later stages contains both genes with a greater sequence divergence from close relatives and younger genes that are more recently evolved. Sequence divergence at later stages in flowering plants probably relates to differences in seed biology, such as dormancy potential, and is unlikely to be constrained by the need to produce a functional adult from an established body plan (Fig. 1). As long as the embryo can still successfully germinate, variation may be selected to aid dispersal and adaptation to germinate in response to different climatic conditions. The transcriptome at this stage also contains evolutionarily younger genes, which is not surprising as seeds are a younger invention, arising approx. 350 million years ago (Mya) (Linkies *et al.*, 2010), compared with plant embryogenesis, that probably evolved as plants became adapted to land between 480 and 420 Mya (Bowman *et al.*, 2007). Indeed embryos in the non-seed plants such as the bryophytes do not undergo this late maturation phase (Harada *et al.*, 2010).

### Developmental robustness and hourglasses

Developmental networks have evolved dramatic robustness to changes in kinetic parameters associated with most system components (von Dassow *et al.*, 2000; Eldar *et al.*, 2002; Meir *et al.*, 2002). Such robustness to uncertainty and noise is considered as a hallmark of living systems (Kitano, 2004) and has been observed in a number of developmental processes (reviewed in MacNeil and Walhout, 2011). The robustness in embryogenesis is thought to rely on the architecture of the gene regulatory networks underlying developmental systems (Lin *et al.*, 2009). It seems likely that robustness is an evolved characteristic, and there is, in many cases, a selective advantage to evolving robust embryonic systems that produce the same outputs in widely varying environmental situations. Given the propensity to evolve robustness and thus constraint, is the waist of the developmental hourglass just a reflection of its age? Does the ‘phylotypic stage’ represent the oldest, most robust, most integrated part of a developmental system? If this were the case, then the convergent evolution of developmental hourglasses in animals and in plants might be seen as inevitable, rather than coincidence. Indeed research has identified developmental hourglasses in other analogous developmental systems (e.g. *Coprinopsis cinerea*) (Cheng *et al.*, 2015).

A transcriptomic hourglass (TAI and TDI) has been observed over the developmental life cycle of the mushroom *C. cinerea* (Cheng *et al.*, 2015). Although *C. cinera* lacks a process of embryogenesis (Kues, 2000), it still exhibits a transcriptomic hourglass waist albeit shifted towards late development (Cheng *et al.*, 2015). This period of the hourglass is characterized by an upregulation of information storage and processing genes, including transcription factors and RNA processing factors, a signature of other developmental networks. The waist of the hourglass is followed by an upregulation of metabolism genes that encode enzymes related to carbohydrate metabolism, which may function to generate osmolytes, synthesize fungal cell wall components and hence support the rapid elongation of the mushroom stems required for spore distribution (Cheng *et al.*, 2015). This observation suggests that the transcriptomic hourglass in *C. cinera* represents a developmental network essential for maintaining developmental outcomes even though it is not coupled with a morphological hourglass pattern (Cheng *et al.*, 2015).

Alongside this, there is some tentative evidence that hourglass patterns may form spontaneously due to the nature of developmental networks. Evolutionary modelling of regulatory gene interactions in simulated hierarchical gene networks can produce hourglass patterns (Akhshabi *et al.*, 2014). This pattern is reflected both in the divergence of gene expression and, interestingly, in ages of genes, with the oldest genes being expressed in the ‘waist of the hourglass’ (Akhshabi *et al.*, 2014). These hourglass networks are produced in situations where developmental regulators have increasingly specific functions (Akhshabi *et al.*, 2014), a situation consistent with the biology of embryos. That simulation of random networks produces hourglass patterns indicates a propensity of all such networks to form this pattern.

### Possible roles for the developmental hourglass model in developmental robustness and species diversity

It seems likely that developmental hourglasses are a common pattern in embryogenesis-like processes, and may even occur spontaneously because of the architecture of transcription factor-driven gene regulation (Akhshabi *et al.*, 2014; Friedlander *et al.*, 2015). Even if these patterns arise spontaneously, there are implications for developmental robustness and species diversity from the constraints that the hourglass places on development. Hourglass structures imply that the waist of the hourglass is the most sensitive region to perturbation in the network. These observations are supported by studies in *C. elegans* where genes expressed during the phylotypic period are known, from loss-of-function studies, to have significant effects on nematode morphology or function (Levin *et al.*, 2012). These data imply that the genes expressed at the waist of the hourglass are highly connected and are conserved due to selection against mutations creating deleterious pleiotropic effects (Artieri, 2012). This suggests that there is selection against mutations/variation in genes expressed during the phylotypic period of the developmental hourglass, thus limiting evolvability in this period. Is it possible that the phylotypic stages of animal and plant embryos represent frozen accidents, developmental networks that have been fixed in structure as newer developmental networks have grown to act before them, in response to new developmental signals, and after them to produce detailed morphology shaped by selection? As these stages become ever more integrated, robust and pleotropic, they become ever more invariant.

Perhaps the frozen phylotypic stages we see today are a reflection, probably highly embroidered, of ancestral networks that allowed the first metazoans and plants to regulate their morphology effectively. This frozen regulation, however, has consequences. The phylotypic stage must constrain the morphological space which an embryo is able to occupy; evolution beyond the phylotypic stage can cause dramatic shifts in morphology, but these changes are still based on the conserved body plan of each phyla, and are conserved by the ancient gene regulatory network lurking in the waist of the hourglass. Whether produced by selection, or as an unforeseen consequence of the evolution of gene regulatory networks, it seems that developmental hourglasses constrain variation in both plants and animals, linking their morphological range and, in turn, species diversity.

## CONCLUSIONS

The phylotranscriptomic hourglass patterns associated with embryogenesis in animals and plants are convergent as embryogenesis evolved independently in animals and plants (Meyerowitz, 2002), but may be a consequence of similar processes in developmental regulation. The hourglass pattern constrains the evolution of embryogenesis, producing a developmental stage that every embryo must pass through with reduced evolutionary, transcriptomic and morphological variation. This stage appears to evolve in both plants and animals, there is evidence for it in fungi and modelling suggests it may occur spontaneously. Despite this, the developmental hourglass must (and does) limit variation, constraining variation that can exist or survive. Variation readily evolves before and after the phylogenetic stage, providing the glorious diversity of plants and animals alive today, but constraint does seem to exist. It is an intriguing thought that the way transcription factors act to regulate gene networks may spontaneously lead to the waist of developmental hourglasses, and that these in turn constrain the morphology that can be produced, or that will be viable. Is the very nature of developmental gene regulation responsible for the production of phyla?

## References

[mcw024-B1] Akam M. 1987 The molecular basis for metameric pattern in the *Drosophila* embryo. Development 101: 1–22. 2896587

[mcw024-B2] Akhshabi S Sarda S Dovrolis C Yi S. 2014 An explanatory evo-devo model for the developmental hourglass. F1000Research 3: 156. doi:10.12688/f1000research.4583.1. 25210617 10.12688/f1000research.4583.1 PMC4156030

[mcw024-B3] Arthur W Farrow M. 1999 The pattern of variation in centipede segment number as an example of developmental constraint in evolution. Journal of Theoretical Biology 200: 183–191. 10504284 10.1006/jtbi.1999.0986

[mcw024-B4] Artieri CG 2012 The rapid evolution of gene expression In: RS Singh J Xu RJ Kulathinal, eds. Rapidly evolving genes and genetic system. Oxford: Oxford University Press, 237–245.

[mcw024-B5] Artieri CG Haerty W Singh RS. 2009 Ontogeny and phylogeny: molecular signatures of selection, constraint, and temporal pleiotropy in the development of *Drosophila*. BMC Biology 7: 42. 19622136 10.1186/1741-7007-7-42 PMC2722573

[mcw024-B6] Bharathan G Goliber TE Moore C Kessler S Pham T Sinha NR. 2002 Homologies in leaf form inferred from KNOXI gene expression during development. Science 296: 1858–1860. 12052958 10.1126/science.1070343

[mcw024-B7] Bininda-Emonds OR Jeffery JE Richardson MK. 2003 Inverting the hourglass: quantitative evidence against the phylotypic stage in vertebrate development. *Proceedings of the* Royal Society B: Biological Sciences 270: 341–346. 10.1098/rspb.2002.2242 PMC1691251 12639312

[mcw024-B8] von Baer KE 1828 Uber Entwickelungsgeschichte der Thiere: Beobachtung und Reflektion. Köenigsberg: Bei den Gebrüdern Bornträger.

[mcw024-B9] Bowman JL Floyd SK Sakakibara K. 2007 Green genes – comparative genomics of the green branch of jife. Cell 129: 229–234. 17448980 10.1016/j.cell.2007.04.004

[mcw024-B10] Brady SM Zhang L Megraw M, 2011 A stele-enriched gene regulatory network in the *Arabidopsis* root. Molecular Systems Biology 7: 459. 21245844 10.1038/msb.2010.114 PMC3049412

[mcw024-B11] Busch A Horn S Muhlhausen A Mummenhoff K Zachgo S. 2012 Corolla monosymmetry: evolution of a morphological novelty in the *Brassicaceae* family. Molecular Biology and Evolution 29: 1241–1254. 22135189 10.1093/molbev/msr297

[mcw024-B12] Busch A Zachgo S. 2009 Flower symmetry evolution: towards understanding the abominable mystery of angiosperm radiation. Bioessays 31: 1181–1190. 19847818 10.1002/bies.200900081

[mcw024-B13] Cameron RA Chow SH Berney K, 2005 An evolutionary constraint: strongly disfavored class of change in DNA sequence during divergence of *cis*-regulatory modules. Proceedings of the National Academy of Sciences, USA 102: 11769–11774. 10.1073/pnas.0505291102 PMC1188003 16087870

[mcw024-B14] Carroll SB. 2008 Evo-devo and an expanding evolutionary synthesis: a genetic theory of morphological evolution. Cell 134: 25–36. 18614008 10.1016/j.cell.2008.06.030

[mcw024-B15] Chan YF Marks ME Jones FC, 2010 Adaptive evolution of pelvic reduction in sticklebacks by recurrent deletion of a Pitx1 enhancer. Science 327: 302–305. 20007865 10.1126/science.1182213 PMC3109066

[mcw024-B16] Chandler J Nardmann J Werr W. 2008 Plant development revolves around axes. Trends in Plant Science 13: 78–84. 18262821 10.1016/j.tplants.2007.11.010

[mcw024-B17] Cheng X Hui JH Lee YY Wan Law PT Kwan HS. 2015 A ‘developmental hourglass’ in fungi. Molecular Biology and Evolution 32: 1556–1566. 25725429 10.1093/molbev/msv047

[mcw024-B18] Comte A Roux J Robinson-Rechavi M. 2010 Molecular signaling in zebrafish development and the vertebrate phylotypic period. Evolution and Development 12: 144–156. 20433455 10.1111/j.1525-142X.2010.00400.x PMC2855863

[mcw024-B19] Cruickshank T Wade MJ. 2008 Microevolutionary support for a developmental hourglass: gene expression patterns shape sequence variation and divergence in *Drosophila*. Evolution and Development 10: 583–590. 18803776 10.1111/j.1525-142X.2008.00273.x

[mcw024-B20] von Dassow G Meir E Munro EM Odell GM. 2000 The segment polarity network is a robust developmental module. Nature 406: 188–192. 10910359 10.1038/35018085

[mcw024-B21] Davidson EH. 2010 Emerging properties of animal gene regulatory networks. Nature 468: 911–920. 21164479 10.1038/nature09645 PMC3967874

[mcw024-B22] Davidson EH Levine MS. 2008 Properties of developmental gene regulatory networks. Proceedings of the National Academy of Sciences, USA 105: 20063–20066. 10.1073/pnas.0806007105 PMC2629280 19104053

[mcw024-B23] Davis JC Brandman O Petrov DA. 2005 Protein evolution in the context of *Drosophila* development. Journal of Molecular Evolution 60: 774–785. 15909223 10.1007/s00239-004-0241-2

[mcw024-B24] Demuth JP Wade MJ. 2007 Maternal expression increases the rate of bicoid evolution by relaxing selective constraint. Genetica 129: 37–43. 16955333 10.1007/s10709-006-0031-4

[mcw024-B25] Domazet-Loso T Tautz D. 2010 A phylogenetically based transcriptome age index mirrors ontogenetic divergence patterns. Nature 468: 815–818. 21150997 10.1038/nature09632

[mcw024-B26] Drost HG Gabel A Grosse I Quint M. 2015 Evidence for active maintenance of phylotranscriptomic hourglass patterns in animal and plant embryogenesis. Molecular Biology and Evolution 32: 1221–1231. 25631928 10.1093/molbev/msv012 PMC4408408

[mcw024-B27] Duboule D. 1994 Temporal colinearity and the phylotypic progression: a basis for the stability of a vertebrate Bauplan and the evolution of morphologies through heterochrony. Development Supplement 1994: 135–142. 7579514

[mcw024-B28] Eldar A Dorfman R Weiss D Ashe H Shilo BZ Barkai N. 2002 Robustness of the BMP morphogen gradient in *Drosophila* embryonic patterning. Nature 419: 304–308. 12239569 10.1038/nature01061

[mcw024-B29] Friedlander T Mayo AE Tlusty T Alon U. 2015 Evolution of bow-tie architectures in biology. PLoS Computer Biology 11: e1004055. doi:10.1371/journal.pcbi.1004055. 10.1371/journal.pcbi.1004055 PMC4370773 25798588

[mcw024-B30] Galis F Metz JA. 2001 Testing the vulnerability of the phylotypic stage: on modularity and evolutionary conservation. Journal of Experimental Zoology Part B: Molecular and Developmental Evolution 291: 195–204. 10.1002/jez.1069 11479918

[mcw024-B31] Garcia-Fernandez J. 2005 Hox, ParaHox, ProtoHox: facts and guesses. Heredity (Edinburgh) 94: 145–152. 15578045 10.1038/sj.hdy.6800621

[mcw024-B32] Gavin-Smyth J Wang YC Butler I Ferguson EL. 2013 A genetic network conferring canalization to a bistable patterning system in *Drosophila*. Current Biology 23: 2296–2302. 24184102 10.1016/j.cub.2013.09.055 PMC4495911

[mcw024-B33] Gifford EM Foster AS 1989 Morphology and evolution of vascular plants. New York: W.H. Freeman and Company.

[mcw024-B34] Goldstein B Frisse LM Thomas WK. 1998 Embryonic axis specification in nematodes: evolution of the first step in development. Current Biology 8: 157–160. 9443914 10.1016/s0960-9822(98)70062-4

[mcw024-B35] Goltsev Y Fuse N Frasch M Zinzen RP Lanzaro G Levine M. 2007 Evolution of the dorsal–ventral patterning network in the mosquito, *Anopheles gambiae*. Development 134: 2415–2424. 17522157 10.1242/dev.02863

[mcw024-B36] van der Graaff E Hooykaas PJ Keller B. 2002 Activation tagging of the two closely linked genes LEP and VAS independently affects vascular cell number. The Plant Journal 32: 819–830. 12472696 10.1046/j.1365-313x.2002.01470.x

[mcw024-B37] Hall BK. 1997 Phylotypic stage or phantom: is there a highly conserved embryonic stage in vertebrates? Trends in Ecology and Evolution 12: 461–463. 21238158 10.1016/s0169-5347(97)01222-6

[mcw024-B38] Harada JJ Belmonte MF Kwong RW 2010 Plant embryogenesis (zygotic and somatic). Chichester, UK: John Wiley and Sons, Ltd.

[mcw024-B39] Harding K Wedeen C McGinnis W Levine M. 1985 Spatially regulated expression of homeotic genes in *Drosophila*. Science 229: 1236–1242. 3898362 10.1126/science.3898362

[mcw024-B40] Hay A Tsiantis M. 2006 The genetic basis for differences in leaf form between *Arabidopsis thaliana* and its wild relative *Cardamine hirsuta*. Nature Genetics 38: 942–947. 16823378 10.1038/ng1835

[mcw024-B41] Hay A Tsiantis M. 2010 KNOX genes: versatile regulators of plant development and diversity. Development 137: 3153–3165. 20823061 10.1242/dev.030049

[mcw024-B42] Hazkani-Covo E Wool D Graur D. 2005 In search of the vertebrate phylotypic stage: a molecular examination of the developmental hourglass model and von Baer’s third law. Journal of Experimental Zoology Part B: Molecular and Developmental Evolution 304: 150–158. 15779077 10.1002/jez.b.21033

[mcw024-B43] Hilgers V Bushati N Cohen SM. 2010 *Drosophila* microRNAs 263a/b confer robustness during development by protecting nascent sense organs from apoptosis. PLoS Biology 8: e1000396. doi:10.1371/journal.pbio.1000396. 20563308 10.1371/journal.pbio.1000396 PMC2885982

[mcw024-B44] Holme P. 2011 Metabolic robustness and network modularity: a model study. PLoS One 6: e16605. doi:10.1371/journal.pone.0016605. 21311770 10.1371/journal.pone.0016605 PMC3032788

[mcw024-B45] ten Hove CA Lu KJ Weijers D. 2015 Building a plant: cell fate specification in the early *Arabidopsis* embryo. Development 142: 420–430. 25605778 10.1242/dev.111500

[mcw024-B46] Irie N Kuratani S. 2011 Comparative transcriptome analysis reveals vertebrate phylotypic period during organogenesis. Nature Communications 2: 248. 10.1038/ncomms1248 PMC3109953 21427719

[mcw024-B47] Irie N Sehara-Fujisawa A. 2007 The vertebrate phylotypic stage and an early bilaterian-related stage in mouse embryogenesis defined by genomic information. BMC Biology 5: 1. 17222327 10.1186/1741-7007-5-1 PMC1797197

[mcw024-B48] Irish VF Martinez-Arias A Akam M. 1989 Spatial regulation of the Antennapedia and Ultrabithorax homeotic genes during *Drosophila* early development. EMBO Journal 8: 1527–1537. 2569971 10.1002/j.1460-2075.1989.tb03537.x PMC400984

[mcw024-B49] Irmler I Schmidt K Starck JM. 2004 Developmental variability during early embryonic development of zebra fish, *Danio rerio*. Journal of Experimental Zoology Part B: Molecular and Developmental Evolution 302: 446–457. 10.1002/jez.b.21010 15580642

[mcw024-B50] Johansen DA 1950 Plant embryology. Waltham, MA: Chronica Botanica Co.

[mcw024-B51] Johri BM Ambegaokar KB Srivastava PS 1992 Comparative embryology of angiosperms. Berlin: Springer-Verlag.

[mcw024-B52] Jurgens G Mayer U Busch M Lukowitz W Laux T. 1995 Pattern formation in the *Arabidopsis* embryo: a genetic perspective. Philosophical Transactions of the Royal Society B: Biological Sciences 350: 19–25. 10.1098/rstb.1995.0132 8577846

[mcw024-B53] Kalinka AT Varga KM Gerrard DT, 2010 Gene expression divergence recapitulates the developmental hourglass model. Nature 468: 811–814. 21150996 10.1038/nature09634

[mcw024-B54] Kitano H. 2004 Biological robustness. Nature Reviews Genetics 5: 826–837. 10.1038/nrg1471 15520792

[mcw024-B55] Kues U. 2000 Life history and developmental processes in the basidiomycete *Coprinus cinereus*. Microbiology and Molecular Biology Reviews 64: 316–353. 10839819 10.1128/mmbr.64.2.316-353.2000 PMC98996

[mcw024-B56] Levin M Hashimshony T Wagner F Yanai I. 2012 Developmental milestones punctuate gene expression in the *Caenorhabditis* embryo. Developmental Cell 22: 1101–1108. 22560298 10.1016/j.devcel.2012.04.004

[mcw024-B57] Li XY MacArthur S Bourgon R, 2008 Transcription factors bind thousands of active and inactive regions in the *Drosophila* blastoderm. PLoS Biology 6: e27. 18271625 10.1371/journal.pbio.0060027 PMC2235902

[mcw024-B58] Liang Z Biggin MD. 1998 Eve and ftz regulate a wide array of genes in blastoderm embryos: the selector homeoproteins directly or indirectly regulate most genes in *Drosophila*. Development 125: 4471–4482. 9778506 10.1242/dev.125.22.4471

[mcw024-B59] Lin KT-H Broitman-Maduro G Hung WWK Cervantes S Maduro MF. 2009 Knockdown of SKN-1 and the Wnt effector TCF/POP-1 reveals differences in endomesoderm specification in *C. briggsae* as compared with *C. elegans*. Developmental Biology 325: 296–306. 18977344 10.1016/j.ydbio.2008.10.001 PMC2648516

[mcw024-B60] Linkies A Graeber K Knight C Leubner-Metzger G. 2010 The evolution of seeds. New Phytologist 186: 817–831. 20406407 10.1111/j.1469-8137.2010.03249.x

[mcw024-B61] MacNeil LT Walhout AJ. 2011 Gene regulatory networks and the role of robustness and stochasticity in the control of gene expression. Genome Research 21: 645–657. 21324878 10.1101/gr.097378.109 PMC3083081

[mcw024-B62] Maduro MF. 2009 Structure and evolution of the *C. elegans* embryonic endomesoderm network. Biochimica et Biophysica Acta 1789: 250–260. 18778800 10.1016/j.bbagrm.2008.07.013 PMC2688470

[mcw024-B63] Maheshwarl P. 1950 An introduction to the embryology of angiosperms. New York: McGraw-Hill.

[mcw024-B64] McGinnis W Krumlauf R. 1992 Homeobox genes and axial patterning. Cell 68: 283–302. 1346368 10.1016/0092-8674(92)90471-n

[mcw024-B65] Meir E von Dassow G Munro E Odell GM. 2002 Robustness, flexibility, and the role of lateral inhibition in the neurogenic network. Current Biology 12: 778–786. 12015114 10.1016/s0960-9822(02)00839-4

[mcw024-B66] de Mendoza A Sebé-Pedrós A Šestak MS, 2013 Transcription factor evolution in eukaryotes and the assembly of the regulatory toolkit in multicellular lineages. Proceedings of the National Academy of Sciences, USA 110: E4858–E4866. 10.1073/pnas.1311818110 PMC3864300 24277850

[mcw024-B67] Meyerowitz EM. 2002 Plants compared to animals: the broadest comparative study of development. Science 295: 1482–1485. 11859185 10.1126/science.1066609

[mcw024-B68] Mordhorst AP Toonen MAJ de Vries SC Meinke D. 1997 Plant embryogenesis. Critical Reviews in Plant Sciences 16: 535–576.

[mcw024-B69] Peel AD Chipman AD Akam M. 2005 Arthropod segmentation: beyond the *Drosophila* paradigm. Nature Reviews Genetics 6: 905–916. 10.1038/nrg1724 16341071

[mcw024-B70] Peris CI Rademacher EH Weijers D. 2010 Green beginnings – pattern formation in the early plant embryo. Current Topics in Developmental Biology 91: 1–27. 20705177 10.1016/S0070-2153(10)91001-6

[mcw024-B71] Peter IS Davidson EH. 2011 Evolution of gene regulatory networks controlling body plan development. Cell 144: 970–985. 21414487 10.1016/j.cell.2011.02.017 PMC3076009

[mcw024-B72] Poethig RS. 1987 Clonal analysis of cell lineage patterns in plant development. American Journal of Botany 74: 581–594.

[mcw024-B73] Pollock EG Jensen WA. 1964 Cell development during early embryogenesis in *Capsella* and *Gossypium*. American Journal of Botany 51: 915–921.

[mcw024-B74] Preston JC Hileman LC. 2009 Developmental genetics of floral symmetry evolution. Trends in Plant Science 14: 147–154. 19231272 10.1016/j.tplants.2008.12.005

[mcw024-B75] Prud’homme B Gompel N Rokas A, 2006 Repeated morphological evolution through *cis*-regulatory changes in a pleiotropic gene. Nature 440: 1050–1053. 16625197 10.1038/nature04597

[mcw024-B76] Quint M Drost HG Gabel A Ullrich KK Bonn M Grosse I. 2012 A transcriptomic hourglass in plant embryogenesis. Nature 490: 98–101. 22951968 10.1038/nature11394

[mcw024-B77] Raff RA. 1996 The shape of life: genes, development and the evolution of animal form. University of Chicago Press.

[mcw024-B78] Raghavan V Sharma KK 1995 Zygotic embryogenesis in Gymnosperms and Angiosperms. Dordrecht: Kluwer Academic Publishers.

[mcw024-B79] Richardson MK. 1995 Heterochrony and the phylotypic period. Development Biology 172: 412–421. 10.1006/dbio.1995.8041 8612960

[mcw024-B80] Richardson MK Hanken J Gooneratne ML, 1997 There is no highly conserved embryonic stage in the vertebrates: implications for current theories of evolution and development. Anatomy and Embryology 196: 91–106. 9278154 10.1007/s004290050082

[mcw024-B81] Roux J Robinson-Rechavi M. 2008 Developmental constraints on vertebrate genome evolution. PLoS Genetics 4: e1000311. doi:10.1371/journal.pgen.1000311. 19096706 10.1371/journal.pgen.1000311 PMC2600815

[mcw024-B82] Sander K. 1975 Pattern specification in the insect embryo. Ciba Foundation Symposia 0: 241–263. 1039911 10.1002/9780470720110.ch12

[mcw024-B83] Schep AN Adryan B. 2013 A comparative analysis of transcription factor expression during metazoan embryonic development. PLoS One 8: e66826. doi:10.1371/journal.pone.0066826. 23799133 10.1371/journal.pone.0066826 PMC3682979

[mcw024-B84] Shubin N Tabin C Carroll S. 2009 Deep homology and the origins of evolutionary novelty. Nature 457: 818–823. 19212399 10.1038/nature07891

[mcw024-B85] Slack JM Holland PW Graham CF. 1993 The zootype and the phylotypic stage. Nature 361: 490–492. 8094230 10.1038/361490a0

[mcw024-B86] Stern DL Orgogozo V. 2008 The loci of evolution: how predictable is genetic evolution? Evolution 62: 2155–2177. 18616572 10.1111/j.1558-5646.2008.00450.x PMC2613234

[mcw024-B87] Stern DL Orgogozo V. 2009 Is genetic evolution predictable? Science 323: 746–751. 19197055 10.1126/science.1158997 PMC3184636

[mcw024-B88] Sucena E Delon I Jones I Payre F Stern DL. 2003 Regulatory evolution of shavenbaby/ovo underlies multiple cases of morphological parallelism. Nature 424: 935–938. 12931187 10.1038/nature01768

[mcw024-B89] Tena JJ González-Aguilera C Fernández-Miñán A, 2014 Comparative epigenomics in distantly related teleost species identifies conserved cis-regulatory nodes active during the vertebrate phylotypic period. Genome Research 24: 1075–1085. 24709821 10.1101/gr.163915.113 PMC4079964

[mcw024-B90] Wardlaw CW. 1955 Embryogenesis in plants. London: Methuen.

[mcw024-B91] Wendrich JR Weijers D. 2013 The *Arabidopsis* embryo as a miniature morphogenesis model. New Phytologist 199: 14–25. 23590679 10.1111/nph.12267

[mcw024-B92] West M Harada JJ. 1993 Embryogenesis in higher plants: an overview. The Plant Cell 5: 1361–1369. 12271035 10.1105/tpc.5.10.1361 PMC160368

[mcw024-B93] Wilson MJ Dearden PK. 2009 Tailless patterning functions are conserved in the honeybee even in the absence of Torso signaling. Development Biology 335: 276–287. 10.1016/j.ydbio.2009.09.002 19735651

[mcw024-B94] Wilson MJ Havler M Dearden PK. 2010 Giant, Kruppel, and caudal act as gap genes with extensive roles in patterning the honeybee embryo. Development Biology 339: 200–211. 10.1016/j.ydbio.2009.12.015 20026025

[mcw024-B95] Yadegari R Goldberg RB. 1997 Embryogenesis in dicotyledonous plants In: BA Larkins IK Vasil, eds. Cellular and molecular biology of plant seed development. Dordrecht: Kluwer Academic Press.

[mcw024-B96] Yanai I Peshkin L Jorgensen P Kirschner MW. 2011 Mapping gene expression in two *Xenopus* species: evolutionary constraints and developmental flexibility. Developmental Cell 20: 483–496. 21497761 10.1016/j.devcel.2011.03.015 PMC3118554

[mcw024-B97] Zheng L Sepulveda LA Lua RC Lichtarge O Golding I Sokac AM. 2013 The maternal-to-zygotic transition targets actin to promote robustness during morphogenesis. PLoS Genetics 9: e1003901. doi:10.1371/journal.pgen.1003901. 24244181 10.1371/journal.pgen.1003901 PMC3820746

